# Sequential cetuximab/bevacizumab therapy is associated with improved outcomes in patients with wild‐type KRAS exon 2 metastatic colorectal cancer

**DOI:** 10.1002/cam4.2235

**Published:** 2019-05-15

**Authors:** Hung‐Chih Hsu, Yu‐Chun Liu, Chuang‐Wei Wang, Wen-Chi Chou, Yu-Jen Hsu, Jy-Ming Chiang, Yung-Chang Lin, Tsai-Sheng Yang

**Affiliations:** ^1^ Division of Hematology‑Oncology Chang Gung Memorial Hospital at Linkou Taoyuan Taiwan; ^2^ College of Medicine Chang Gung University Taoyuan Taiwan; ^3^ Department of Dermatology, Drug Hypersensitivity Clinical and Research Center Chang Gung Memorial Hospitals Taipei Taiwan; ^4^ Chang Gung Immunology Consortium, Chang Gung Memorial Hospital and Chang Gung University Taoyuan Taiwan; ^5^ Division of Colon and Rectal Surgery Chang Gung Memorial Hospital at Linkou Taoyuan Taiwan

**Keywords:** anti‐EGFR/anti‐VEGF, biological therapy sequence, metastatic colorectal cancer, survival

## Abstract

**Purpose:**

Combination of biological therapy and chemotherapy improves the survival of patients with metastatic colorectal cancer (mCRC). However, the optimal biological therapy sequence remains unclear. In this retrospective study, we evaluated the clinical outcomes of patients with mCRC treated with different sequences of biological therapies as first‐ and third‐line therapy.

**Methods:**

We only included patients with wild‐type KRAS exon 2 mCRC who had received cetuximab, bevacizumab, and standard chemotherapy. The patients were treated with cetuximab or bevacizumab as first‐ or third‐line therapy combined with a similar chemotherapy backbone.

**Results:**

In total, 102 patients were included. Forty‐six patients received first‐line cetuximab therapy followed by third‐line bevacizumab therapy (cetuximab → bevacizumab group) and 56 patients received first‐line bevacizumab therapy followed by third‐line cetuximab therapy (bevacizumab → cetuximab group). The cetuximab → bevacizumab group was associated with increased survival (OS) compared with the bevacizumab → cetuximab group (median OS: 30.4 months vs 25.7 months, hazard ratio (HR): 0.55, 95% confidence interval (CI): 0.36‐0.86). When calculated from the start of second‐ and third‐line therapies, OS was also higher in the cetuximab → bevacizumab group (second‐line: 20.6 months vs 14.8 months, HR: 0.54, 95% CI: 0.34‐0.81; third‐line: 12.5 months vs 9.9 months, HR: 0.53, 95% CI: 0.35‐0.83). The cetuximab → bevacizumab group was also associated with better progression‐free survival than the bevacizumab → cetuximab group (8.8 vs 4.5 months, HR: 0.43, 95% CI: 0.25‐0.58) in the third‐line setting, but not in the first‐ or second‐line settings.

**Conclusions:**

Our study demonstrated that first‐line cetuximab therapy followed by third‐line bevacizumab therapy was associated with favorable clinical outcomes as compared to the reverse sequence.

## 1 INTRODUCTION

Colorectal cancer (CRC) is one of the most common cancer types and is a leading cause of cancer death worldwide.^1^ Despite improvements in therapies (including drugs and surgery), half of CRC patients still develop metastatic disease, which is a major cause of death and contributor to the low 5‐year survival rate.^2^ Recent advances in therapy for metastatic CRC (mCRC), such as the addition to standard chemotherapy of biological therapies targeting angiogenesis or proliferation pathways, have increased the overall survival (OS) of patients with mCRC to 24‐30 months and beyond.^3^, ^4^, ^5^, ^6^, ^7^

Combination treatment with cytotoxic chemotherapy agents (5‐fluorouracil [FU], oxaliplatin, and irinotecan) and targeted therapies (anti‐epidermal growth factor receptor [EGFR; panitumumab and cetuximab] and anti‐vascular endothelial growth factor [VEGF; bevacizumab] monoclonal antibodies) has been the standard therapy for mCRC. Anti‐VEGF antibody as first‐line therapy, second‐line therapy, or therapy beyond progression has been shown to improve the survival of patients with mCRC.^8^, ^9^, ^10^, ^11^, ^12^ Anti‐EGFR antibody as first‐ or third‐line therapy has also been reported to improve survival.^13^, ^14^, ^15^

Although different sequences of these biological therapies can be provided to patients, the optimal sequence remains unknown. A phase III study (FIRE3 study) revealed that, in patients with wild‐type KRAS exon 2 or RAS mCRC, the combination of anti‐EGFR antibody (cetuximab) with chemotherapy as first‐line treatment provided higher OS than the combination of bevacizumab with chemotherapy.^5^ Another phase II study in which another anti‐EGFR antibody (panitumumab) was combined with chemotherapy showed similar results.^6^ Furthermore, subgroup analysis suggested that the OS improvement resulting from first‐line anti‐EGFR therapy may be the impact of subsequent later‐line therapies and the sequence of targeted therapies.^16^, ^17^ Several retrospective studies have also indicated that cetuximab ( after bevacizumab failure may decrease the efficacy of cetuximab.^18^, ^19^, ^20^ However, a phase III trial (the CALGB study) comparing cetuximab‐based first‐line regimens with bevacizumab‐based first‐line regimens showed contradictory results; that is, no differences were observed in OS or progression‐free survival (PFS) between the two regimens.^21^ The imbalance and diversity of later‐line therapy in the FIRE3 study are critical issues that affect OS, instead of the therapy sequence.^22^, ^23^

In this retrospective study, to determine whether the therapy sequence affects OS, we enrolled patients with wild‐type KRAS exon 2 mCRC who had received cetuximab, bevacizumab, and three cytotoxic chemotherapy agents. This study evaluated the effects of different sequences of biological therapy on the clinical outcomes of patients with wild‐type KRAS exon 2 mCRC under a similar and balanced chemotherapy backbone setting.

## 2 MATERIALS AND METHODS

### 2.1 Study population

We retrospectively reviewed the medical records of patients diagnosed with mCRC at the Chang Gung Memorial Hospital in Linkou, Taiwan, between July 2012 and December 2016. Patients were included if they had: (a) histologically proven colorectal adenocarcinoma; (b) undergone anti‐VEGF therapy (bevacizumab), anti‐EGFR therapy (cetuximab), and treatment with three chemotherapy drugs (5‐FU, oxaliplatin, and irinotecan); (c) wild‐type KRAS exon 2 mCRC; and (d) measurable lesions before starting therapy. This study was approved by the Chang Gung Memorial Hospital Institutional Review Board (IRB 201601421B0) and was performed in accordance with the Declaration of Helsinki. The requirement for informed consent was waived for this study.

### 2.2 Therapy and clinical characteristics

Demographic and clinical characteristics were obtained through a review of the patients’ medical records. These characteristics included gender, age, tumor histological grade, primary tumor location, metastatic site and organ, stage, performance status, therapy record (including drug and surgery), and clinical outcome.

In Taiwan, according to the National Health Insurance program, biological therapy (bevacizumab or cetuximab) should be combined with irinotecan‐based chemotherapy. Therefore, in this study, the chemotherapy backbone was irinotecan‐based or oxaliplatin‐based, and the sequence of therapy was irinotecan‐based therapy as first‐line therapy followed by oxaliplatin‐ and irinotecan‐based therapy as second‐line and third‐line therapies. Regarding the biological therapy sequence, all patients received either first‐line cetuximab therapy followed by third‐line bevacizumab therapy or first‐line bevacizumab therapy followed by third‐line cetuximab therapy. The irinotecan‐based regimens included irinotecan and infusional 5‐FU with leucovorin (FOLFIRI) (leucovorin 400 mg/m^2^ iv over 2 h before 5‐FU on day 1, 5‐FU 400 mg/m^2^ iv bolus on day 1 and 2400 mg/m^2^ iv over 46 hours, and irinotecan 180 mg/m^2^ iv over 90 min on day 1 every 2 weeks),^24^ irinotecan and bolus 5‐FU with leucovorin (IFL) (leucovorin 20 mg/m^2^ iv bolus qw × 4 weeks every 6 weeks, 5‐FU 500 mg/m^2^ iv bolus qw × 4 weeks every 6 weeks, and irinotecan 125 mg/m^2^ iv qw × 4 weeks every 6 weeks),^25^ and irinotecan only.^26^ The oxaliplatin‐based regimen included oxaliplatin and infusional 5‐FU with leucovorin (mFOLFOX6) (leucovorin 400 mg/m^2^ iv over 2 hours before 5‐FU on day 1, 5‐FU 400 mg/m^2^ iv bolus on day 1 followed by 2400 mg/m^2^ iv over 46 hours, and oxaliplatin 85 mg/m^2^ iv over 2 hours on day 1 every 2 weeks).^27^ None of the patients received biological therapy as second‐line therapy.

### 2.3 Definitions of second‐ and third‐line therapy

Second‐line therapy was defined as the administration of any anticancer drug that was not included in the first‐line regimen. Third‐line therapy was defined as the administration of any anticancer drug that was not included in the second‐line regimen.

### 2.4 Clinical outcome assessment

Responses to therapy were evaluated using computed tomography (CT) based on Response Evaluation Criteria in Solid Tumors (RECIST) criteria (version 1.1).^28^ Radiological examination (CT) was performed routinely every 12‐16 weeks depending on the respective chemotherapy protocol. The responses included complete response (CR), partial response (PR), stable disease (SD), and progressive disease (PD). We also calculated the objective response rate (ORR) and disease control rate (DCR). PFS was calculated from the starting date of first‐line therapy to disease progression (radiologic or clinical progression) or death from any cause. OS was calculated from the starting date of first‐line therapy to death from any cause. PFS after first‐line therapy (first‐line PFS) was defined as the date of the first application of first‐line therapy to disease progression (radiologic or clinical progression) or death from any cause. PFS after second‐line therapy (second‐line PFS) was defined as the date of the first application of second‐line therapy to disease progression (radiologic or clinical progression) or death from any cause. PFS after third‐line therapy (third‐line PFS) was defined as the date of the first application of third‐line therapy to disease progression (radiologic or clinical progression) or death from any cause. Second‐line OS was defined as the date of the first application of second‐line therapy to death from any cause. Third‐line OS was defined as the date of the first application of third‐line therapy to death from any cause. PFS, OS, first‐line PFS, second‐line PFS, third‐line PFS, second‐line OS, and third‐line OS are expressed as medians.

### 2.5 Statistical analysis

PFS, OS, first‐line PFS, second‐line PFS, third‐line PFS, second‐line OS, and third‐line OS were estimated using the Kaplan‐Meier method and log‐rank test. Between‐group comparisons of variables (including therapy duration) were performed using the *t* test, chi‐square, or Fisher exact test. Hazard ratios (HRs) were calculated using Cox regression analysis and are presented with their 95% confidence intervals (CIs). All statistical tests were two‐sided, and *P* < 0.05 was considered statistically significant. All statistical analyses were performed using GraphPad Prism (version 7.0; GraphPad Inc, San Diego, CA) and IBM SPSS software (version 20; SPSS, Chicago, IL).

## 3 RESULTS

### 3.1 Patient characteristics

This study only included 102 patients with wild‐type KRAS exon 2 mCRC, and their characteristics are listed in Table 1. Of the 102 patients, 46 received first‐line cetuximab/FOLFIRI therapy followed by third‐line bevacizumab/irinotecan‐based therapy (cetuximab → bevacizumab group), and 56 received first‐line bevacizumab/FOLFIRI therapy followed by third‐line cetuximab/irinotecan‐based therapy (bevacizumab → cetuximab group). All patients received mFOLFOX6 chemotherapy as second‐line therapy. Third‐line therapy details are listed in Table 2 Forty patients (39%) received FOLFIRI chemotherapy combined with cetuximab or bevacizumab (19 patients with bevacizumab in the cetuximab → bevacizumab group and 21 patients with cetuximab in the bevacizumab → cetuximab group), 39 patients (38%) received IFL chemotherapy combined with cetuximab or bevacizumab (27 patients with bevacizumab in the cetuximab → bevacizumab group and 12 patients with cetuximab in the bevacizumab → cetuximab group), and 23 (23%) received irinotecan combined with cetuximab (all in the bevacizumab → cetuximab group). Baseline characteristics were well‐balanced between the two groups. The median age was 56 years. Most patients were synchronous mCRC (71%). The liver was the most frequent metastatic site. Moreover, 44 patients with synchronous mCRC underwent resection of the primary colorectal tumor, and 61 received therapy beyond third‐line therapy. The average follow‐up duration was 28.6 months (standard deviation: 11.4 months, range: 8.9‐64.3 months).

**Table 1:** Characteristics of patients with metastatic colorectal cancer

	All patients (N (%))	Cetuximab → Bevacizumab (N (%))	Bevacizumab → cetuximab (N (%))	*P*‐value^a^
Total number	102	46	56	
Sex				1.000
Male	62 (61)	28 (63)	34 (59)	
Female	40 (39)	18 (37)	22 (41)	
Median (range)	56 (31‐83)	54 (31‐83)	57 (31‐81)	
Age				0.557
≤56	52 (51)	25 (54)	27 (48)	
>56	50 (49)	21 (46)	29 (52)	
Metastatic pattern				1.000
Metachronous	30 (29)	14 (30)	16 (29)	
Synchronous	72 (71)	32 (70)	40 (71)	
ECOG PS^b^				0.503
0	86 (84)	40 (87)	46 (82)	
1	16 (16)	6 (13)	16 (18)	
Tumor Histology				1.000
Low grade^c^	89 (87)	40 (87)	49 (88)	
High grade^c^	13 (13)	6 (13)	7 (12)	
Primary tumor location				
Right side^d^	13 (13)	6 (13)	7 (12)	1.000
Left side^d^	89 (87)	40 (87)	49 (88)	
Colon	58 (57)	29 (63)	29 (52)	0.316
Rectum	44 (43)	17 (37)	27 (48)	
Metastatic site				
Liver	61	26	35	
Lung	35	17	18	
Other	44	21	33	
Number of metastatic sites				0.840
1	60 (59)	28 (61)	32 (57)	
>1	42 (41)	18 (39)	24 (43)	
Primary CRC tumor resection in synchronous mCRC (72 pts)^e^				0.331
Primary CRC tumor resection	44 (61)	22 (69)	22 (55)	
No primary CRC tumor resection	28 (39)	10 (31)	18 (45)	
Post‐progression therapy (94 pts)				0.828
Postprogression therapy(yes)	61 (65)	24 (63)	37 (66)	
Postprogression therapy(no)	33 (35)	14 (37)	19 (34)	

Left side: tumor originating in the splenic flexure, descending colon, sigmoid colon, or rectum.

a Fisher exact *P*‐value.

b ECOG: Eastern Cooperative Oncology Group; PS: Performance Status.

c Low grade: well/moderate‐differentiated; High grade: poor‐differentiated/mucinous/ signet ring cell.

d Right side: tumor originating in the appendix, cecum, ascending colon, hepatic flexure, or transverse colon.

e CRC: colorectal cancer; mCRC: metastatic colorectal cancer.

**Table 2:** Third‐line regimen (biological therapy combined with chemotherapy in two groups)

Therapy (No (%))	Biological therapy sequence	
	Cetuximab → bevacizumab	Bevacizumab → cetuximab
Biological therapy	Bevacizumab (46 (100))	Cetuximab (56 (100))
Chemotherapy		
FOLFIRI	19 (41)	21 (38)
IFL	27 (59)	12 (21)
Irinotecan	0 (0)	23 (41)

Abbreviations: FOLFIRI: irinotecan and infusional 5‐fluorouracil with leucovorin; IFL: irinotecan and bolus 5‐fluorouracil with leucovorin

### 3.2 Efficacy

The DCRs were comparable between the cetuximab → bevacizumab and bevacizumab → cetuximab groups across different lines of therapy (first‐, second‐, and third‐line therapy: 81% vs 77%, 41% vs 52%, and 57% vs 41%, respectively; Table 3). As shown in Table 3, in the first‐line therapy setting, the cetuximab → bevacizumab group showed a higher ORR (70% vs 59%) and metastasectomy rate (19.6% vs 7.1%). The cetuximab → bevacizumab group had a significantly longer duration of third‐line therapy than the bevacizumab → cetuximab group (8.8 vs 5.3 months, *P* = 0.002; Table 3).

**Table 3:** Efficacy and duration of different line therapy

Response/duration		Biological therapy sequence		
		Cetuximab → bevacizumab	Bevacizumab → cetuximab	*P* value^a^
Response (No (%))				
First line	CR/PR	32 (70)	33 (59)	0.305
	SD	5 (11)	10 (18)	
	PD	9 (19)	13 (23)	
	DCR	46 (81)	43 (77)	0.810
	Metastaectomy	9 (19.6)	4 (7.1)	0.058
Second line	CR/PR	12 (26)	6 (11)	0.066
	SD	7 (15)	23 (41)	
	PD	27 (59)	27 (48)	
	DCR	19 (41)	29 (52)	0.324
Third line	CR/PR	10 (22)	13 (23)	1.000
	SD	16 (35)	10 (18)	
	PD	20 (43)	33 (59)	
	DCR	26 (57)	23 (41)	0.163
Duration (months)				*P* value^b^
First line		9.7	9.6	0.920
Second line		6.3	5.1	0.147
Third line		8.8	5.2	0.002

Abbreviations: CR: complete response, PR: partial response, SD: stable disease, DCR: disease control rate.

a Fisher exact test.

b *t* test.

### 3.3 Comparison of survival across different lines of therapy

**Figure 1:**
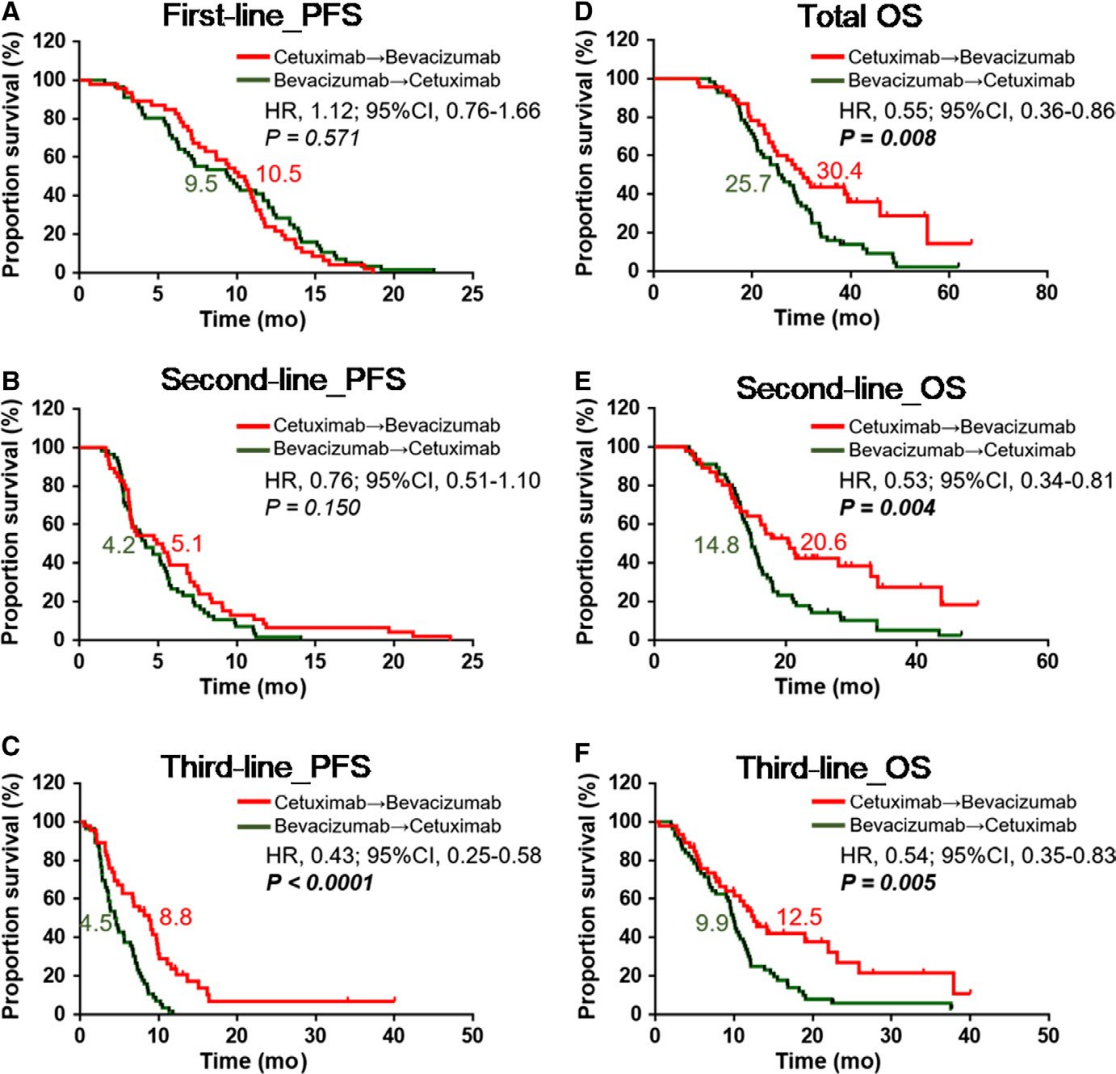
Kaplan‐Meier curves for survival among two kinds of therapy sequence groups in different line therapy. (A‐C). Progression‐free survival (PFS) of two groups with different biological therapy sequence in first‐, second‐, and third‐line therapy. (D‐F). Overall survival (OS) of two groups with different biological therapy sequence in first‐ (total OS), second‐, and third‐line therapy

Total OS was higher in the cetuximab → bevacizumab group than in the bevacizumab → cetuximab group (median OS: 30.4 months vs 25.7 months, HR: 0.55, 95% CI: 0.36 to 0.86, *P* = 0.008, Figure 1D). The second‐line OS and third‐line OS were also higher in the cetuximab → bevacizumab group than in the bevacizumab → cetuximab group (second‐line OS: 20.6 months vs 14.8 months, HR: 0.53, 95% CI: 0.34 to 0.81, *P* = 0.004; third‐line OS: 12.5 months vs 9.9 months, hazard ratio: 0.54, 95% CI: 0.35 to 0.83, *P* = 0.005; Figure 1E,F**)**. Regarding PFS, the cetuximab → bevacizumab group had a higher third‐line PFS than the bevacizumab → cetuximab group (8.8 months vs 4.5 months, HR: 0.43, 95% CI: 0.25 to 0.58, *P* < 0.0001; Figure 1C**)**. However, there were no significant differences between the two groups in first‐line PFS or second‐line PFS (Figure 1A,B).

### 3.4 Subgroup analysis of OS and third‐line OS/PFS

**Figure 2:**
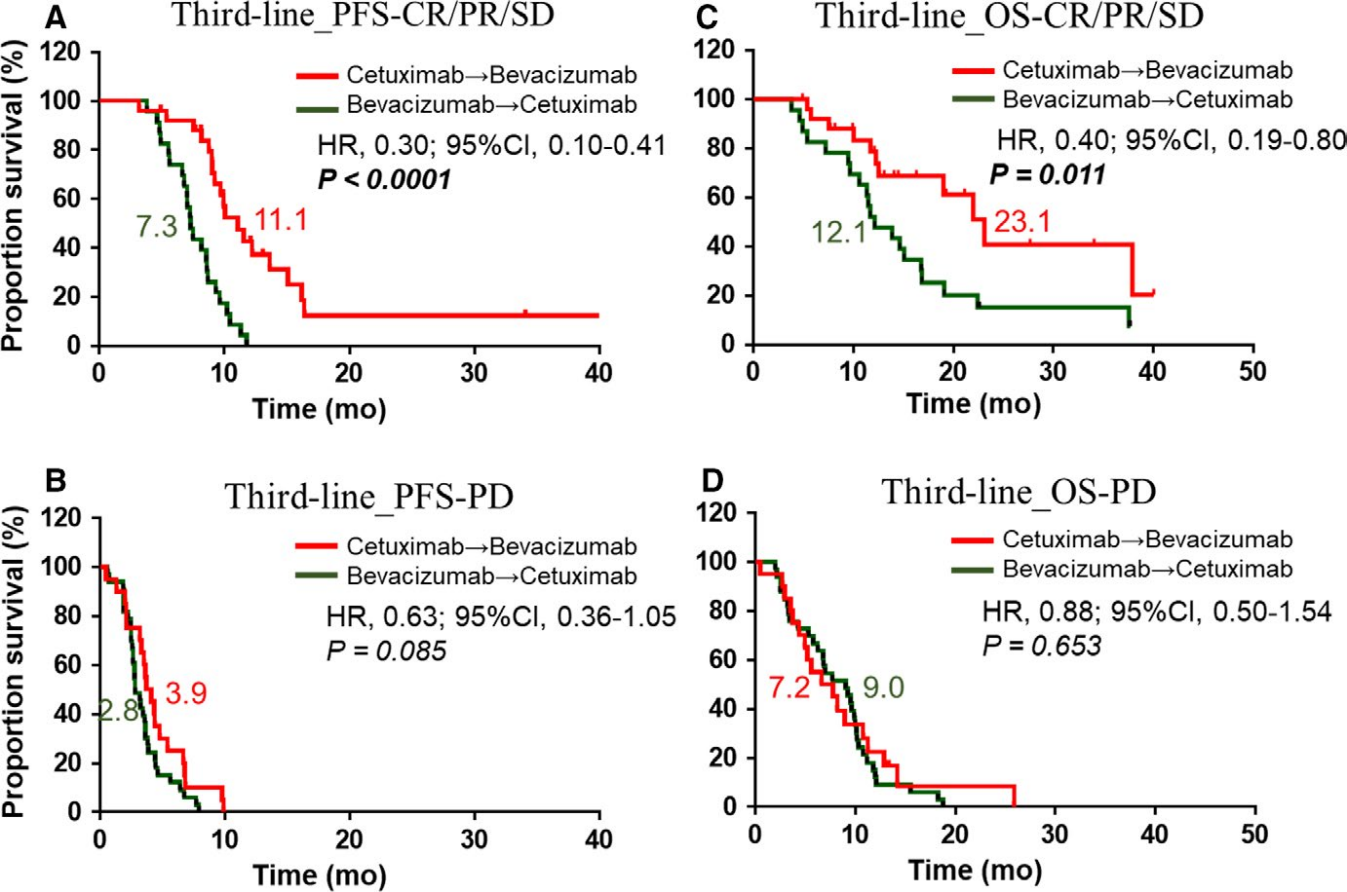
Kaplan‐Meier curves for survival among subgroup in third‐line therapy. (A and B) Progression‐free survival (PFS)/overall survival (OS) of CR/PR/SD group between two different therapy sequences in third‐line therapy. (C and D) Progression‐free survival (PFS)/overall survival (OS) of PD group between two different therapy sequences in third‐line therapy. Abbreviations: CR: complete response; PR: partial response; SD: stable disease; PD: progressive disease

**Figure 3:**
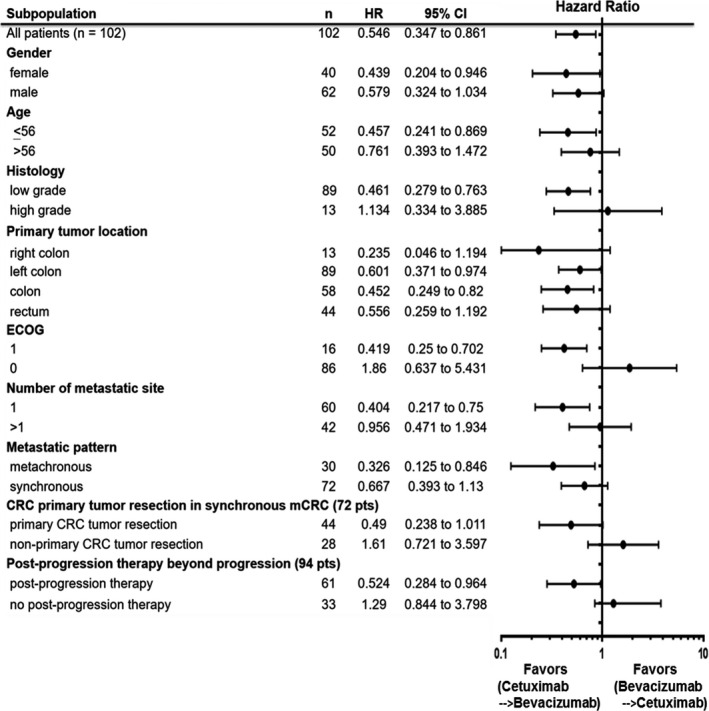
Forest plot of treatment hazard ratios (HRs) with 95% CIs for overall survival in each subgroup

In the third‐line therapy setting, among patients with CR, PR, or SD, patients in the cetuximab → bevacizumab group had a higher OS and PFS than those in the bevacizumab → cetuximab group (third‐line OS: 23.1 months vs 12.1 months, HR: 0.40, 95% CI: 0.19 to 0.80, *P* < 0.011; third‐line PFS: 11.1 months vs 7.3 months, HR: 0.30; 95, CI: 0.10 to 0.41, *P* < 0.0001, Figure 2A,2). There was no significant difference in survival between the two groups among patients with PD (Figure 2B,D). In subgroup analysis of OS, in most subpopulations, the biological therapy sequence of cetuximab → bevacizumab resulted in higher survival, which was similar to what we observed in the entire population (Figure 3).

## 4 DISCUSSION

We performed this study to compare the clinical outcomes of patients with wild‐type KRAS exon 2 mCRC who received two targeted therapies (ie, anti‐EGFR and anti‐VEGF antibodies) and standard chemotherapy (with three chemotherapy drugs) based on the sequence of biological therapy. The results demonstrated that administration of anti‐EGFR antibody before anti‐VEGF antibody improved OS compared with administration of anti‐VEGF antibody before anti‐EGFR antibody. They also showed that the survival benefit from third‐line biological therapy contributed to improved OS. This is the first study to demonstrate that the sequence of anti‐EGFR/anti‐VEGF antibodies as first‐ and third‐line therapy may determine the OS and affect the clinical outcome of third‐line biological therapy in patients with wild‐type KRAS exon 2 mCRC.

In this study, the cetuximab → bevacizumab group had higher OS than the bevacizumab → cetuximab group. Favorable OS was also seen in the cetuximab → bevacizumab group in most subgroups, and OS was statistically higher in patients aged <56 years, women, and patients with tumors in the left colon, low histologic grade cancer, or metachronous cancer. The cetuximab → bevacizumab group also had longer OS than the bevacizumab → cetuximab group among patients with right‐sided tumors with marginal significance (HR: 95% CI:, *P =* 0.061), which is contradictory to the relationship between sidedness and EGFR first‐line treatment efficacy elucidated in several randomized trials.^29^ This might be attributable to selection bias in our study, in that patients with right‐sided colon tumors that had higher survival received all three cytotoxic chemotherapy drugs and two biological drugs (anti‐EGFR/anti‐VEGF antibodies) across three lines of therapy, which is uncommon in patients with right‐sided colon tumors in large randomized studies.^29^ In the patients who received postprogression therapy, the cetuximab → bevacizumab sequence resulted in longer OS. Similar to the FIRE‐3 trial,^5^ the patients receiving cetuximab as first‐line therapy had higher response and metastasectomy rates. In this study, approximately 20% of the patients demonstrated an objective response to third‐line biological therapy, which is comparable to the proportions reported in previous studies of third‐line therapy.^13^, ^30^, ^31^

The OS was higher in the cetuximab → bevacizumab group than in the bevacizumab → cetuximab group across different lines of therapy. However, only third‐line PFS was better in the cetuximab → bevacizumab group. This result suggests that the selection of cetuximab (anti‐EGFR therapy) as first‐line therapy in combination with chemotherapy for mCRC is associated with increased survival of third‐line bevacizumab therapy. The efficacy of third‐line therapy, including the DCR and ORR, were similar between the two groups. However, the duration of SD after third‐line therapy was significantly longer in the cetuximab → bevacizumab group (8.78 vs 5.16 months, *P =* 0.002), which might lead to the significant difference in third‐line PFS between the two groups.

Extended RAS (KRAS and NRAS, exons 2‐4) analysis has been performed in clinical practice since November 2015. In our cohort, KRAS exon 2 analysis was performed in all patients (102 patients) and extended RAS analysis in only about a third of patients (32 patients). Moreover, the RAS result may not represent our true RAS result because of the small sample size.

Similar to our study, the Prodige 18 study^32^ and several retrospective studies^18^, ^19^ have also demonstrated that prior bevacizumab therapy decreases the efficacy of subsequent cetuximab therapy. Although Modest et al reported that the application of anti‐EGFR therapy as first‐line therapy may represent a favorable condition for promoting the effectiveness of subsequent antiangiogenic agents,^16^ the diversity of second‐line therapy and imbalance of later‐line regimens contributed to the uncertainness of this result. In this study, we reduced the imbalance of subsequent therapy; hence, it was clear that first‐line cetuximab therapy affected later‐line bevacizumab therapy and increased OS in patients with wild‐type KRAS exon 2 mCRC.

Preclinical studies in animal models and cancer cell lines have shown that after resistance to EGFR inhibitors was developed, constitutively high expression of VEGF, vascular endothelial growth factor receptor‐1(VEGFR‐1), and placental growth factor (PlGF) were noted, and inhibition of the VEGF pathway by VEGF inhibitor treatment was shown to be effective.^33^, ^34^, ^35^, ^36^ In contrast, after bevacizumab pretreatment, hypoxia was induced in cancer cell lines, and subsequent EGFR‐independent RAS activation rendered the cells less sensitive to subsequent EGFR blockade.^18^, ^37^, ^38^, ^39^, ^40^ These phenomena might explain this study results; bevacizumab as third‐line therapy leads to prolonged duration of SD because this therapy alters the tumor microenvironment through the inhibition of VEGF/VEGFR. Conversely, cetuximab as third‐line therapy contributes to shorter duration of SD because of EGFR‐independent RAS signal activation.

The results of this study may affect patients with initially unresectable mCRC, who may receive all three cytotoxic chemotherapy drugs and two biological therapy drugs (anti‐EGFR/anti‐VEGF antibodies) across three lines of therapy in a palliative therapy setting. For this group, our data could help optimize the biological sequence in clinical practice, such that the sequence of anti‐EGFR antibody followed by anti‐VEGF antibody leads to better OS and clinical outcome from later‐line biological therapy.

This study results indicate that, in addition to chemotherapy with various drugs, selecting the optimal biological therapy sequence is important for improving clinical outcomes in palliative drug therapy for mCRC. However, there were several limitations of our study, including the small sample size, retrospective single‐institution nature, lack of more extended RAS test in all of the patients, and selection bias of therapy, which depended on the physician’s decision in clinical practice. Further validation with prospective multicenter studies is required. The ongoing GERCOR STRATEGIC‐1 study, which is an international, open‐label, randomized, multicenter phase III trial, is designed to give global information on the therapeutic sequences in patients with unresectable RAS wild‐type mCRC and is likely to have a significant impact on the management of this patient population.

## 5 CONCLUSION

This study demonstrated that administration of anti‐EGFR antibody followed by anti‐VEGF antibody was associated with better OS and clinical outcomes of biological therapy in later lines than administration of anti‐VEGF antibody followed by anti‐EGFR antibody. It provided the information how to optimize the biological sequence in clinical practice.

## CONFLICTS OF INTEREST

The authors declare that they have no conflict of interest.

## AUTHORSHIP

Hung‐Chih Hsu: concept and design, writing of the manuscript, data interpretation, analysis, and acquisition; Yu‐Chun Liu: aid in writing manuscript, data interpretation, and acquisition; Chuang‐Wei Wang: aid in writing manuscript, data interpretation, and analysis; Wen‐Chi Chou, Yu‐Jen Hsu, Jy‐Ming Chiang, and Yung‐Chang Lin: data analysis and acquisition; Tsai‐Sheng Yang: concept and design, supervision, data interpretation, and analysis.

## Data Availability

The data that support the findings of this study are available on request from the corresponding author. The data are not publicly available due to privacy or ethical restrictions.

## References

[1] Torre LA , Bray F , Siegel RL , Ferlay J , Lortet‐Tieulent J , Jemal A . Global cancer statistics, 2012. CA Cancer J Clin. 2015;65(2):87‐108. 10.3322/caac.21262. 25651787

[2] Welch HG , Robertson DJ . Colorectal cancer on the decline‐why screening can't explain it all. N Engl J Med. 2016;374(17):1605‐1607. 10.1056/NEJMp1600448. 27119236

[3] Van Cutsem E , Cervantes A , Adam R , et al. ESMO consensus guidelines for the management of patients with metastatic colorectal cancer. Ann Oncol. 2016;27:1386‐1422. 27380959 10.1093/annonc/mdw235

[4] Wainberg ZA , Drakaki A . The importance of optimal drug sequencing in metastatic colorectal cancer: biological rationales for the observed survival benefit conferred by first‐line treatment with EGFR inhibitors. Expert Opin Biol Ther. 2015;15:1205‐1220. 26066903 10.1517/14712598.2015.1050375

[5] Heinemann V , von Weikersthal LF , Decker T , et al. FOLFIRI plus cetuximab versus FOLFIRI plus bevacizumab as first‐line treatment for patients with metastatic colorectal cancer (FIRE‐3): a randomised, open‐label, phase 3 trial. Lancet Oncol. 2014;15:1065‐1075. 25088940 10.1016/S1470-2045(14)70330-4

[6] Schwartzberg LS , Rivera F , Karthaus M , et al. PEAK: a randomized, multicenter phase II study of panitumumab plus modified fluorouracil, leucovorin, and oxaliplatin (mFOLFOX6) or bevacizumab plus mFOLFOX6 in patients with previously untreated, unresectable, wild‐type KRAS exon 2 metastatic colorectal cancer. J Clin Oncol. 2014;32:2240‐2247. 24687833 10.1200/JCO.2013.53.2473

[7] Venook AP , Niedzwiecki D , Lenz HJ , et al. Effect of first‐line chemotherapy combined with cetuximab or bevacizumab on overall survival in patients with KRAS wild‐type advanced or metastatic colorectal cancer: A randomized clinical trial. JAMA. 2017;317:2392‐2401. 28632865 10.1001/jama.2017.7105 PMC5545896

[8] Giantonio BJ , Catalano PJ , Meropol NJ , et al. Bevacizumab in combination with oxaliplatin fluorouracil, and leucovorin (FOLFOX4) for previously treated metastatic colorectal cancer: results from the eastern cooperative oncology group study E3200. J Clin Oncol. 2007;25(12):1539‐1544. 17442997 10.1200/JCO.2006.09.6305

[9] Van Cutsem E , Tabernero J , Lakomy R , et al. Addition of aflibercept to fluorouracil, leucovorin, and irinotecan improves survival in a phase III randomized trial in patients with metastatic colorectal cancer previously treated with an oxaliplatin‐based regimen. J Clin Oncol. 2012;30(28):3499‐3506. 22949147 10.1200/JCO.2012.42.8201

[10] Tabernero J , Van Cutsem E , Lakomý R , et al. Aflibercept versus placebo in combination with fluorouracil, leucovorin and irinotecan in the treatment of previously treated metastatic colorectal cancer: prespecified subgroup analyses from the VELOUR trial. Eur J Cancer. 2014;50(2):320‐331. 24140268 10.1016/j.ejca.2013.09.013

[11] Bennouna J , Sastre J , Arnold D , et al. Continuation of bevacizumab after first progression in metastatic colorectal cancer (ML18147): a randomised phase 3 trial. Lancet Oncol. 2013;14(1):29‐37. 23168366 10.1016/S1470-2045(12)70477-1

[12] Tabernero J , Yoshino T , Cohn AL , et al. Ramucirumab versus placebo in combination with second‐line FOLFIRI in patients with metastatic colorectal carcinoma that progressed during or after first‐line therapy with bevacizumab, oxaliplatin, and a fluoropyrimidine (RAISE): a randomised, double‐blind, multicentre, phase 3 study. Lancet Oncol. 2015;16(5):499‐508. 25877855 10.1016/S1470-2045(15)70127-0

[13] Cunningham D , Humblet Y , Siena S , et al. Cetuximab monotherapy and cetuximab plus irinotecan in irinotecan‐refractory metastatic colorectal cancer. N Engl J Med. 2004;351:337‐345. 15269313 10.1056/NEJMoa033025

[14] Douillard JY , Rong A , Sidhu R . RAS mutations in colorectal cancer. N Engl J Med. 2013;369:2159‐2160. 10.1056/NEJMc1312697 24283232

[15] Van Cutsem E , Köhne CH , Hitre E , et al. Cetuximab and chemotherapy as initial treatment for metastatic colorectal cancer. N Engl J Med. 2009;360:1408‐1417. 19339720 10.1056/NEJMoa0805019

[16] Modest DP , Stintzing S , von Weikersthal LF , et al. Impact of subsequent therapies on outcome of the FIRE‐3/AIO KRK0306 trial: first‐line therapy with FOLFIRI plus Cetuximab or Bevacizumab in patients with KRAS wild‐type tumors in met‐ astatic colorectal cancer. J Clin Oncol. 2015;33(32):3718‐3726. 26261259 10.1200/JCO.2015.61.2887

[17] Peeters M , Forget F , Karthaus M , et al. Exploratory pooled analysis evaluating the effect of sequence of biological therapies on overall survival in patients with *RAS* wild‐type metastatic colorectal carcinoma. ESMO Open. 2018;3(2):e000297. 29531837 10.1136/esmoopen-2017-000297 PMC5844379

[18] Derangère V , Fumet JD , Boidot R , et al. Does bevacizumab impact anti‐EGFR therapy efficacy in metastatic colorectal cancer? Oncotarget. 2016;7(8):9309‐9321. 26824184 10.18632/oncotarget.7008 PMC4891042

[19] Sato Y , Matsusaka S , Suenaga M , Shinozaki E , Mizunuma N . Cetuximab could be more effective without prior bevacizumab treatment in metastatic colorectal cancer patients. Onco Targets Ther. 2015;11(8):3329‐3336. 10.2147/OTT.S89241 PMC4648607 26648737

[20] Norguet E , Dahan L , Gaudart J , Gasmi M , Ouafik L , Seitz JF . Cetuximab after bevacizumab in metastatic colorectal cancer: is it the best sequence? Dig Liver Dis. 2011;43(11):917‐919. 21763224 10.1016/j.dld.2011.06.002

[21] Venook AP , Niedzwiecki D , Lenz HJ , et al. CALGB/SWOG 80405: phase III trial of irinotecan/5‐FU/leucovorin (FOLFIRI) or oxaliplatin/5‐FU/leucovorin (mFOLFOX6) with bevacizumab (BV) or cetuximab (CET) for patients (pts) with KRAS wild‐type (wt) untreated metastatic adenocarcinoma of the colon or rectum (MCRC). Proc Am Soc. Clin Oncol. 2014;32:LBA3.

[22] Avallone A , Budillon A . Impact of subsequent therapies on outcome of the FIRE‐3/AIO KRK0306 trial. J Clin Oncol. 2016;34(13):1564. 26951321 10.1200/JCO.2015.66.1512

[23] Nasti G , Ottaiano A . Best sequence therapy in RAS wild‐type metastatic colorectal cancer: waiting for randomized crossover clinical trials. J Clin Oncol. 2016;34(13):1563‐1564. 26951319 10.1200/JCO.2015.65.9086

[24] Tournigand C , André T , Achille E , et al. FOLFIRI followed by FOLFOX6 or the reverse sequence in advanced colorectal cancer: a randomized GERCOR study. J Clin Oncol. 2004;15;22(2):229‐237. 10.1200/JCO.2004.05.113 14657227

[25] Saltz LB , Cox JV , Blanke C , et al. Irinotecan plus fluorouracil and leucovorin for metastatic colorectal cancer. N Engl J Med. 2000;343(13):905‐911. 11006366 10.1056/NEJM200009283431302

[26] Vincenzi B , Santini D , Rabitti C , et al. Cetuximab and irinotecan as third‐line therapy in advanced colorectal cancer patients: a single centre phase II trial. Br J Cancer. 2006;94(6):792‐797. 16508634 10.1038/sj.bjc.6603018 PMC2361373

[27] Cheeseman SL , Joel SP , Chester JD , et al. A 'modified de Gramont' regimen of fluorouracil, alone and with oxaliplatin, for advanced colorectal cancer. Br J Cancer. 2002;87(4):393‐399. 12177775 10.1038/sj.bjc.6600467 PMC2376131

[28] Eisenhauer EA , Therasse P , Bogaerts J , et al. New response evaluation criteria in solid tumours: Revised RECIST guideline (version 1.1). Eur J Cancer. 2009;45(2):228‐247. 19097774 10.1016/j.ejca.2008.10.026

[29] Arnold D , Lueza B , Douillard JY , et al. Prognostic and predictive value of primary tumour side in patients with RAS wild‐type metastatic colorectal cancer treated with chemotherapy and EGFR directed antibodies in six randomized trials. Ann Oncol. 2017;28(8):1713‐1729. 28407110 10.1093/annonc/mdx175 PMC6246616

[30] Yang Q , Yin C , Liao F , et al. Bevacizumab plus chemotherapy as third‐ or later‐line therapy in patients with heavily treated metastatic colorectal cancer. Onco Targets Ther. 2015;1(8):2407‐2413. 10.2147/OTT.S88679 PMC4562721 26366095

[31] Kiss I , Bortlicek Z , Melichar B , et al. Efficacy and toxicity of bevacizumab on combination with chemotherapy in different lines of treatment for metastatic colorectal carcinoma. Anticancer Res. 2014;34(2):949‐954. 24511038

[32] Hiret S , Borg C , Bertaut A , et al. Bevacizumab or cetuximab plus chemotherapy after progression with bevacizumab plus chemotherapy in patients with wtKRAS metastatic colorectal cancer: A randomized phase II study (Prodige 18 –UNICANCER GI). J Clin Oncol. 2016;34(15_suppl):3514‐3514. Abstract 3514.

[33] Zhang X , Gaspard JP , Chung DC . Regulation of vascular endothelial growth factor by the Wnt and K‐ras pathways in colonic neoplasia. Cancer Res. 2001;61(16):6050‐6054. 11507052

[34] Bianco R , Rosa R , Damiano V , et al. Vascular endothelial growth factor receptor‐1 contributes to resistance to anti‐epidermal growth factor receptor drugs in human cancer cells. Clin Cancer Res. 2008;14(16):5069‐5080. 18694994 10.1158/1078-0432.CCR-07-4905

[35] Ciardiello F , Bianco R , Caputo R , et al. Antitumor activity of ZD6474, a vascular endothelial growth factor receptor tyrosine kinase inhibitor, in human cancer cells with acquired resistance to antiepidermal growth factor receptor therapy. Clin Cancer Res. 2004;10(2):784‐793. 14760102 10.1158/1078-0432.ccr-1100-03

[36] Viloria‐Petit A , Crombet T , Jothy S , et al. Acquired resistance to the antitumor effect of epidermal growth factor receptor‐blocking antibodies in vivo: a role for altered tumor angiogenesis. Cancer Res. 2001;61(13):5090‐6101. 11431346

[37] Zeng M , Kikuchi H , Pino MS , Chung DC . Hypoxia activates the K‐ras proto‐oncogene to stimulate angiogenesis and inhibit apoptosis in colon cancer cells. PLoS ONE. 2010;5(6):e10966. 20532039 10.1371/journal.pone.0010966 PMC2881039

[38] Stefanini MO , Wu F , Mac Gabhann F , Popel AS . Increase of plasma VEGF after intravenous administration of bevacizumab is predicted by a pharmacokinetic model. Cancer Res. 2010;70(23):9886‐9894. 21118974 10.1158/0008-5472.CAN-10-1419 PMC3058319

[39] Kopetz S , Hoff PM , Morris JS , et al. Phase II trial of infusional fluorouracil, irinotecan, and bevacizumab for metastatic colorectal cancer: efficacy and circulating angiogenic biomarkers associated with therapeutic resistance. J Clin Oncol. 2010;28(3):453‐459. 20008624 10.1200/JCO.2009.24.8252 PMC2815707

[40] Zaniboni A , The FV . The Best. First. Anti‐EGFR before anti‐VEGF, in the first‐line treatment of RAS wild‐typemetastatic colorectal cancer: from bench to bedside. Cancer Chemother Pharmacol. 2016;78(2):233‐244. 27091467 10.1007/s00280-016-3032-8

